# Erratum: Leino-Arjas et al. Process of Work Disability: From Determinants of Sickness Absence Trajectories to Disability Retirement in a Long-Term Follow-Up of Municipal Employees. *Int. J. Environ. Res. Public Health* 2021, *18,* 2614

**DOI:** 10.3390/ijerph18136917

**Published:** 2021-06-28

**Authors:** Päivi Leino-Arjas, Jorma Seitsamo, Clas-Håkan Nygård, Prakash K.C., Subas Neupane

**Affiliations:** 1 Finnish Institute of Occupational Health, 00250 Helsinki, Finland; Paivi.Leino-Arjas@ttl.fi (P.L.-A.); jseitsamo@gmail.com (J.S.); 2 Unit of Health Sciences, Faculty of Social Sciences, Tampere University, FI-33014 Tampere, Finland; clas-hakan.nygard@tuni.fi (C.-H.N.); prakash.kc@tuni.fi (P.K.C.); 3 Gerontology Research Center, Tampere University, FI-33014 Tampere, Finland; 4 Tampere University Hospital, 33521 Tampere, Finland

The authors wish to make the following correction to this paper [1]. While the estimates presented in the text on page 12, where Table 4 is cited, are estimates for a 10-year follow-up and according to our study aim, the included table erroneously presented estimates for a 15-year follow-up. Therefore, the authors wish to replace it with that on a 10-year follow-up as follows:

## Figures and Tables

**Table 4:** Membership in the 5-year sickness absence trajectories (1981–1985) as a predictor of disability pension retirement over a 10-year (1986–1996) follow-up. Cox regression analysis. Hazard ratios (HR) and their 95 % confidence intervals (CI) adjusted for age (model 1) and age and socioeconomic status ^1^ (model 2).

Sickness Absence Trajectories 1981–1985	Model 1		Model 2	
	HR	95% CI	HR	95% CI
**Women**				
Low	1	-	1	-
Intermediate	3.19	2.74–3.71	3.05	2.62–3.56
Increasing	12.7	8.20–19.7	11.8	7.57–18.3
**Men**				
Low	1	-	1	-
Intermediate	2.10	1.80–2.46	2.04	1.74–2.34
Increasing	11.4	7.71–16.7	10.5	7.1–15.5

^1^ basic education and family income.
